# The Scientific Benefits of a Statewide, Standardized, Coastal Wetland Monitoring Program in Hawaiʻi

**DOI:** 10.1002/ece3.71293

**Published:** 2025-04-21

**Authors:** Judith Z. Drexler, Helen Raine, Carrie L. Harrington, Kawika B. Winter, Kaʻuaʻoa Fraiola, Joy Browning, Jeff Burgett, David A. Burney, Kim Falinski, Scott Fisher, Kristen C. Harmon, Jessica L. Idle, Monica Iglecia, Mari‐Vaughn V. Johnson, Matthew Keir, K. Jackson Letchworth, Kirsten Moy, Anthony Olegario, Melissa R. Price, J. Michael Reed, Yoshimi M. Rii, Rachel Rounds, Charles B. van Rees, Bret Wolfe

**Affiliations:** ^1^ California Water Science Center U.S. Geological Survey Sacramento California USA; ^2^ Pacific Birds Habitat Joint Venture Kauaʻi Hawaiʻi USA; ^3^ U.S. Fish and Wildlife Service Honolulu Hawaiʻi USA; ^4^ Department of Natural Resources & Environmental Management University of Hawaiʻi at Mānoa Honolulu Hawaiʻi USA; ^5^ Hawaiʻi Institute of Marine Biology University of Hawaiʻi at Mānoa Heʻeia Hawaiʻi USA; ^6^ Pacific Islands Climate Adaptation Science Center U.S. Geological Survey Honolulu Hawaiʻi USA; ^7^ Makauwahi Cave Reserve Kauaʻi Hawaiʻi USA; ^8^ The Nature Conservancy Hawaiʻi and Palmyra Chapter Honolulu Hawaiʻi USA; ^9^ Hawaiʻi Land Trust Honolulu Hawaiʻi USA; ^10^ Hawaiian Islands Conservation Collective Kailua Hawaiʻi USA; ^11^ Pacific Birds Habitat Joint Venture Portland Oregon USA; ^12^ U.S. Geological Survey, Pacific Islands Climate Adaptation Science Center Hilo Hawaiʻi USA; ^13^ Division of Forestry and Wildlife, Hawaiʻi Department of Land and Natural Resources Honolulu Hawaiʻi USA; ^14^ National Park Service Kailua‐Kona Hawaiʻi USA; ^15^ Hawaiʻi Coral Reef Initiative University of Hawaiʻi at Mānoa Honolulu Hawaiʻi USA; ^16^ Division of Aquatic Resources Hawaiʻi Department of Land and Natural Resources Honolulu Hawaiʻi USA; ^17^ Department of Biology Tufts University Medford Massachusetts USA; ^18^ Odum School of Ecology University of Georgia Athens Georgia USA

**Keywords:** adaptive management, agricultural wetlands, climate change, coastal wetlands, endangered waterbirds, environmental monitoring, loʻi kalo, sea‐level rise

## Abstract

In this viewpoint, we provide a scientific justification for a statewide, standardized, coastal wetland monitoring program for Hawaiʻi, USA. Hawaiian coastal wetlands provide important habitat for endangered waterbirds, invertebrates, plants, and the Hawaiian hoary bat (ʻōpeʻapeʻa; *Lasiurus semotus*) as well as support Indigenous food systems. Currently, numerous agencies and groups in Hawaiʻi collect data on coastal wetlands, but information is not typically shared and methods are not standardized. A statewide, standardized, coastal wetland monitoring program with a centralized database would allow managers to keep better track of progress toward restoration goals, population changes of conservation‐reliant species, outbreaks and impacts of avian botulism, rates of coastal salinization, and many other critical issues across space and time. Monitoring combined with targeted research could fill critical knowledge gaps about the types, functions, values, and biodiversity of Hawaiian coastal wetlands. Ultimately, the improved knowledge gained from long‐term coastal wetland monitoring could inform landscape‐scale restoration actions and adaptive management of coastal wetlands under sea‐level rise and climate change.

## Graphical abstract

Coastal wetlands in Hawaiʻi provide habitat for endangered waterbirds, invertebrates, plants, and the Hawaiian hoary bat, as well as support Indigenous food systems. Establishment of a statewide, coordinated coastal wetland monitoring program could track the recovery of endangered species and improve management of natural and agricultural wetlands under climate change and sea‐level rise.

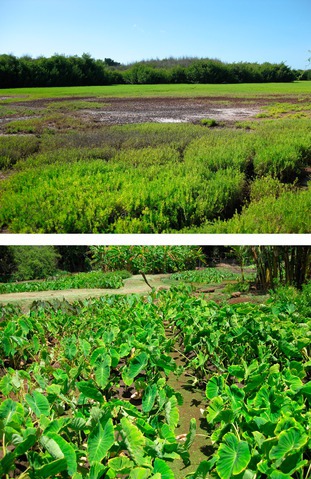

## 1 Introduction

**FIGURE 1:**
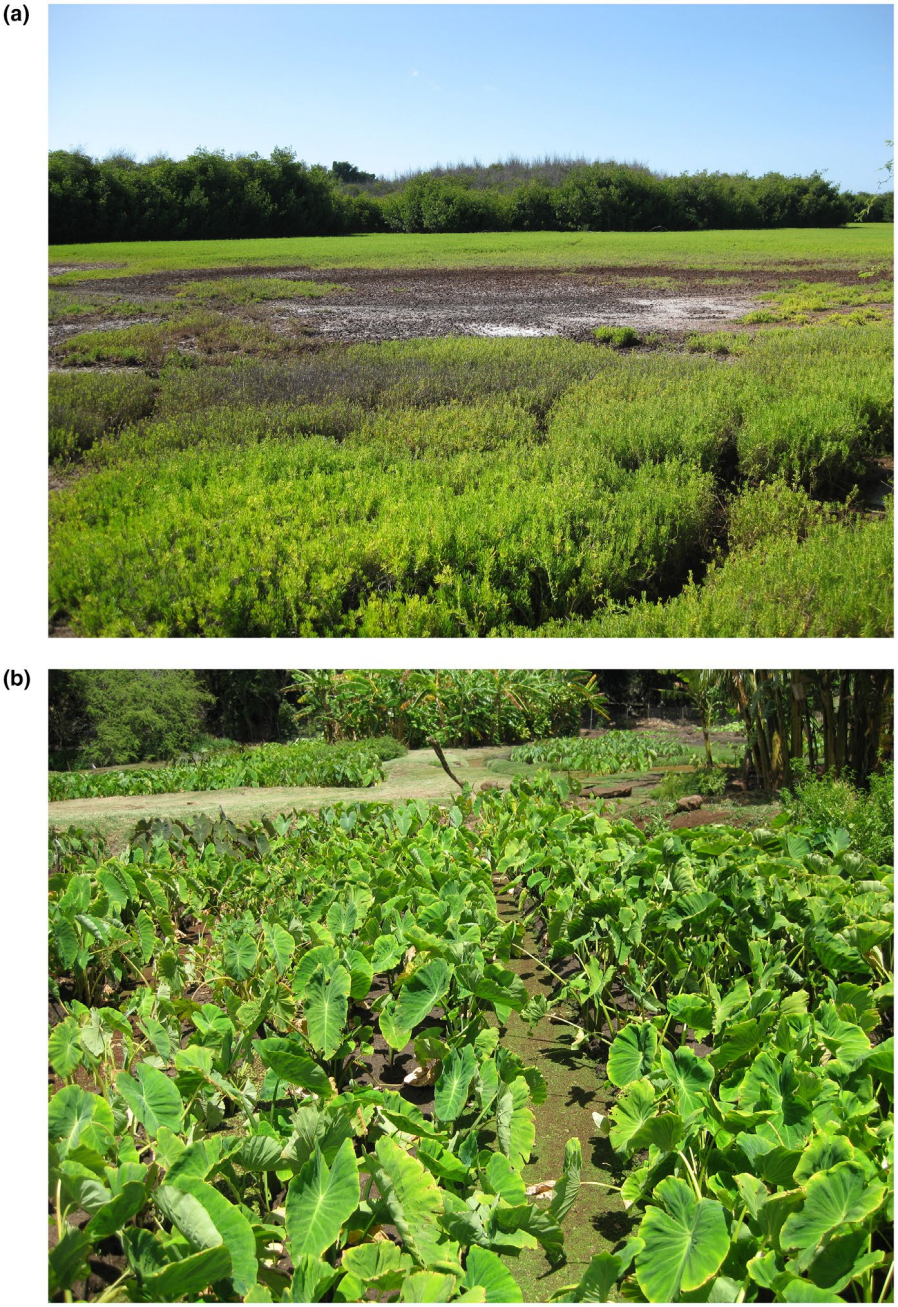
Coastal wetlands along the south shore of Molokaʻi, Hawaiʻi: (a) Punalau Pond I, an herbaceous coastal wetland containing non‐native pickleweed ( *Batis maritima* ) in the foreground and non‐native red mangrove ( *Rhizophora mangle* ) in the background, and (b) Kaupapaloʻi o Kaʻamola, a spring‐fed wetland agro‐ecosystem (loʻipūnāwai), containing taro ( *Colocasia esculenta* ) (photographs by Drexler, U.S. Geological Survey).

Approximately 44% of Hawaiian wetlands situated along the lowland coastal plain (hereafter, coastal wetlands) were converted to rice, pineapple, and sugar plantations following European settlement (Burney et al. 2001; van Rees and Reed 2014). Most of the remaining approximately 212 km^2^ of coastal wetlands have been degraded by non‐native species, development, hydrologic alteration, erosion, and/or sedimentation (Figure 1a; Burney 2002; US Fish and Wildlife Service (USFWS) 2011a; Jacobi and Stock 2017). Despite their degraded condition, coastal wetlands provide habitat for several endemic, threatened or endangered (under the US Endangered Species Act) animals including the orangeblack Hawaiian damselfly (pinapinao in Hawaiian; *Megalagrion xanthomelas* ), Hawaiian stilt (aeʻo; *Himantopus mexicanus knudseni* ), Hawaiian coot (ʻalae keʻokeʻo; *Fulica alai* ), Hawaiian common gallinule (ʻalae ʻula; *Gallinula galeata sandvicensis*), Hawaiian duck (koloa maoli; *Anas wyvilliana* ), Laysan duck (koloa pōhaka; *Anas laysanensis* ), Hawaiian goose (nēnē; *Branta sandvicensis* ), and Hawaiian hoary bat (ʻōpeʻapeʻa; *Lasiurus semotus*; USFWS 2004, 2009, 2011a, 2011b, 2011c, 2016, 2024; Endangered Species Recovery Committee and State of Hawaiʻi Department of Land and Natural Resources (DLNR) Division of Forestry and Wildlife (DOFAW) 2024; Gorresen et al. 2024). Hawaiian coastal wetlands also contain endangered plants including *Marsilea villosa* (ʻihiʻihi), *Cyperus trachysanthos* (puʻukaʻa), and *Ischaemum byrone* (Hilo ischaemum) (Gagné and Cuddihy 1999; Erickson and Puttock 2006; Lichvar et al. 2016).

Coastal wetlands are not comprehensively monitored for their status and trends in Hawaiʻi even though they provide numerous ecosystem services including supporting Indigenous food systems, providing habitat for biodiversity, reducing coastal flooding, and improving water quality (De Groot et al. 2006; Winter et al. 2018). Instead, each organization or agency monitors according to its own mission and domain. For example, the USFWS monitors *M. xanthomelas* , endangered waterbirds, and some additional plant and invertebrate species on federal wildlife refuges and state protected wetlands (USFWS 2011a, 2024). The DLNR monitors insects, mollusks, plants, and fish species of concern on state lands (DLNR Division of Aquatic Resources 2024; DLNR DOFAW 2024a, 2024b, 2024c). DLNR coordinates the biannual State Waterbird Counts on federal, state, and private lands. In addition, the Heʻeia National Estuarine Research Reserve (NERR), a partnership among federal, state, and community‐based entities, monitors water quality, biodiversity, land use and habitat change, and wetland surface elevation (Winter et al. 2020; Heʻeia NERR 2024). Except for State Waterbird Counts, each monitoring effort has its own methodologies, which limit interoperability, collaboration, and data sharing.

## 2 Monitoring Is Critical for Managing Wetlands in a Changing World

Standardized, comprehensive regional monitoring programs are integral to wetland management across the US (e.g., Chesapeake Bay Program 2024; Coastal Wetlands Planning, Protection, and Restoration Act Program 2024; Great Lakes Coastal Wetland Monitoring Program 2024). Access to data on vegetation, sensitive species, soils, land use, elevation, water quality, and hydrology at the regional or statewide level allows wetland managers to view issues at multiple temporal and spatial scales. Such monitoring data can be critical for decision‐making, including how to enhance wetland ecosystem services (Drexler et al. 2019; Windham‐Myers et al. 2023), meet restoration targets at landscape scales (Uzarski et al. 2019; Henkel et al. 2023), and gauge the future resilience of wetlands under sea‐level rise (Raposa et al. 2016; Wasson et al. 2019).

Currently, there are major data gaps regarding Hawaiian coastal wetlands that monitoring and targeted research could address. The biodiversity of plants and invertebrates remains poorly understood (Erickson and Puttock 2006; DNLR, 2024b, 2024c). Data are sparse on basic ecosystem properties including plant productivity, soil characteristics, nutrient status, salinity, and hydrology as well as ecosystem processes including carbon cycling, soil vertical accretion rates, wetland surface elevation change, and decomposition rates (Hill 1996; Erickson and Puttock 2006; Henry 2006; Bantilan‐Smith et al. 2009). Greater knowledge of ecosystem processes could improve adaptive management approaches such as using coastal wetland restoration as a nature‐based climate solution to reduce coastal flooding and increase carbon storage. Furthermore, many properties and processes described above are required inputs for wetland sustainability models, which can provide metrics of wetland degradation and project wetland drowning under sea‐level rise (Ganju et al. 2017; Fagherazzi et al. 2020; Morris et al. 2022). Such modeling can guide adaptive management by determining where to restore freshwater flows or apply thin‐layer sediment placement to wetlands losing ground to sea‐level rise (Myszewski and Alber 2017; Moritsch et al. 2022). Finally, knowledge is also limited on the different types of coastal wetlands in Hawaiʻi. Recent research on the island of Molokaʻi melded Indigenous Knowledge with conventional science to identify a coastal fen, a groundwater‐fed peatland not previously documented in Hawaiian wetland inventories (Drexler et al. 2023).

## 3 Restoration Success Relies on Consistent, Long‐Term Monitoring

There is strong support for ecological restoration in the Native Hawaiian (Kānaka ʻŌiwi) community and conservation groups across the state (Gon et al. 2018; Gon and Winter 2019; Winter et al. 2020; Drexler et al. 2023). Of particular interest is the restoration of Indigenous food systems including loko iʻa (aquaculture systems), loʻi kalo (wetland agro‐ecosystems), loʻi paʻakai (saltbeds), and the kahawai (stream or river) and/or ocean processes that support them (Figure 1b; Sproat 2011; Gon et al. 2018; Harmon et al. 2021). There is also strong interest in restoring natural wetlands and loʻi kalo, using best management practices, to provide habitat for endangered waterbirds and improve coastal resilience along shorelines (Harmon et al. 2022; Raine et al. 2024). Long‐term monitoring, including kilo (Indigenous observations), is critical for tracking progress toward restoration goals, improving restoration techniques, and scaling restoration benefits across the greater landscape (Buchsbaum and Wigand 2012; Morishige et al. 2018; Uzarski et al. 2019). A standardized, statewide monitoring program could provide the spatial and temporal framework for tracking restoration projects while also providing the necessary background data for adaptively managing coastal wetlands under climate change and sea‐level rise. Establishment of a centralized database to coordinate wetland monitoring data could facilitate data sharing about restoration and many other issues including management of conservation‐reliant species, outbreaks of avian botulism, and rates of coastal groundwater salinization.

The process of creating a statewide, standardized, coastal wetland monitoring program would benefit from strong, sustained engagement with restoration and conservation partners and close collaboration with the Kānaka ʻŌiwi community. The importance of Hawaiian coastal wetlands to numerous endangered species, the broad interest in re‐establishing Indigenous food systems, and the critical need to increase coastal resiliency under sea‐level rise together present a compelling case to begin the work as soon as possible.

## Author Contributions

**Judith Z. Drexler:** conceptualization (lead), writing – original draft (lead), writing – review and editing (lead). **Helen Raine:** conceptualization (equal), writing – original draft (equal), writing – review and editing (equal). **Carrie L. Harrington:** conceptualization (equal), writing – original draft (equal), writing – review and editing (equal). **Kawika B. Winter:** conceptualization (equal), writing – original draft (equal), writing – review and editing (equal). **Kaʻuaʻoa Fraiola:** conceptualization (equal), writing – original draft (equal), writing – review and editing (equal). **Joy Browning:** conceptualization (supporting), writing – original draft (supporting), writing – review and editing (supporting). **Jeff Burgett:** conceptualization (supporting), writing – original draft (equal), writing – review and editing (equal). **David A. Burney:** conceptualization (supporting), writing – original draft (equal), writing – review and editing (equal). **Kim Falinski:** conceptualization (supporting), writing – original draft (supporting), writing – review and editing (supporting). **Scott Fisher:** conceptualization (supporting), writing – original draft (supporting), writing – review and editing (equal). **Kristen C. Harmon:** conceptualization (supporting), writing – original draft (supporting), writing – review and editing (supporting). **Jessica L. Idle:** conceptualization (supporting), writing – original draft (supporting), writing – review and editing (supporting). **Monica Iglecia:** conceptualization (supporting), writing – original draft (equal), writing – review and editing (equal). **Mari‐Vaughn V. Johnson:** conceptualization (supporting), writing – original draft (supporting), writing – review and editing (equal). **Matthew Keir:** conceptualization (supporting), writing – original draft (supporting), writing – review and editing (supporting). **K. Jackson Letchworth:** conceptualization (supporting), writing – original draft (supporting), writing – review and editing (equal). **Kirsten Moy:** conceptualization (supporting), writing – original draft (supporting), writing – review and editing (supporting). **Anthony Olegario:** conceptualization (supporting), writing – original draft (supporting), writing – review and editing (supporting). **Melissa R. Price:** conceptualization (supporting), writing – original draft (supporting), writing – review and editing (supporting). **J. Michael Reed:** conceptualization (supporting), writing – original draft (equal), writing – review and editing (equal). **Yoshimi M. Rii:** conceptualization (supporting), writing – original draft (supporting), writing – review and editing (supporting). **Rachel Rounds:** conceptualization (supporting), writing – original draft (supporting), writing – review and editing (supporting). **Charles B. van Rees:** conceptualization (supporting), writing – original draft (equal), writing – review and editing (equal). **Bret Wolfe:** conceptualization (supporting), writing – original draft (supporting), writing – review and editing (supporting).

## Conflicts of Interest

The authors declare no conflicts of interest.

## Data Availability

There were no data collected for this viewpoint.
